# Clinicopathological and prognostic analysis of PIK3CA mutated invasive breast cancer in Chinese women

**DOI:** 10.1016/j.clinsp.2025.100702

**Published:** 2025-06-04

**Authors:** Juhang Chu, Luyao Huang, Yaru Wang, Mingping Qian

**Affiliations:** a Department of General Surgery, Shanghai Tenth People’s Hospital, Tongji University School of Medicine, Shanghai, China; b Tongji University School of Medicine, Shanghai, China

**Keywords:** PIK3CA protein, Breast cancer, Human, Prognostic factors, Nomogram

## Abstract

**Objective:**

To explore the relationship between PIK3CA mutations and clinicopathological features and prognosis in breast cancer patients.

**Methods:**

This retrospective cohort study included 283 patients with invasive breast cancer who underwent surgery from June 2017 to June 2022. PIK3CA exon 9 and 20 mutations were detected using PCR and sequencing. Patients were divided into recurrence (n = 47) and non-recurrence (n = 236) groups based on follow-up data to identify postoperative recurrence factors and build a prediction model. Kaplan-Meier analysis was used to evaluate recurrence rates.

**Results:**

PIK3CA mutations were identified in 41% of patients, with exon 20 mutations being the most common (62.1%). Mutations correlated with tumor quadrant, histological subtype, clinical stage, perineural invasion, and high NLR (p < 0.05). Recurrence group patients showed higher BMI, multiple tumors, non-luminal types, advanced clinical stage, lymph node metastasis, and higher NLR (p < 0.05). Multivariate analysis identified high BMI, multiple tumors, lymph node metastasis, advanced clinical stage, and perineural invasion as independent recurrence risk factors (p < 0.05). Notably, no significant difference in disease-free survival was observed between PIK3CA mutant and wild-type patients (p > 0.05).

**Conclusion:**

PIK3CA mutations are associated with specific clinicopathological features but do not significantly impact prognosis in breast cancer patients.

## HIGHLIGHTS

• PIK3CA mutations involve exon 20 and 9 in breast cancer.
• PIK3CA mutations are linked to tumor characteristics and NLR.
• A tumor recurrence prediction model was established based tumor characteristics.

## Introduction

Breast cancer is a malignant tumor originating in the breast, which seriously threatens the physical and mental health and life safety of women.^1^ In recent years, due to the continuous update of biological cytology and the rapid development of molecular theory and technology, it has been found that gene mutation plays an important role in the occurrence and development of breast cancer.^2^ Phosphoinositide-3-Kinase (PI3K) pathway is the hub of cell growth and downstream metabolic signals of Human Epidermal Growth Factor Receptor 2 (HER2).^3^ PI3K catalytic alpha polypeptidegene, PIK3CA, as an important member of the PI3K family, has been found to have a high frequency of point mutations in various malignant tumors such as colon cancer,^4^ stomach cancer,^5^ neck squamous cell carcinoma^6^ and ovarian cancer,^7^ and is one of the most mutation-prone oncogenes discovered so far. Although PIK3CA mutations are present in all breast cancer subtypes, Estrogen Receptor (ER) positive and HER2 negative patients have a higher mutation frequency.^8^ This variability underscores the importance of considering molecular subtypes when investigating the role of PIK3CA mutations in breast cancer. The clinical relevance of PIK3CA mutations extends beyond their diagnostic utility. These mutations have been implicated in influencing the efficacy of targeted therapies and endocrine treatments. For instance, in HR-positive breast cancers, PIK3CA mutations are associated with resistance to endocrine therapies such as tamoxifen and aromatase inhibitors. Conversely, the advent of PI3K inhibitors, such as alpelisib,^9^ has shown promise in overcoming this resistance and improving outcomes in patients with PIK3CA-mutated breast cancers.

Although the detection of gene mutation status has been used as an important therapeutic strategy in the clinical treatment of a variety of tumors, the relationship between PIK3CA mutations and clinicopathological characteristics and prognosis of breast cancer patients is still controversial. In this regard, this study detected the mutation status of PIK3CA in breast cancer tumor tissues by PCR amplification and DNA sequencing, calculated the mutation incidence, analyzed the relationship between different mutation types and pathological characteristics and prognostic effects of breast cancer as well as analyzed the recurrence of breast cancer after surgery. The authors proposed that high Body Mass Index (BMI), multiple masses, lymph node metastasis, advanced clinical stage and tumor perineural invasion may be independent risk factors for postoperative recurrence of breast cancer, in order to provide relevant reference for clinical treatment of breast cancer.

## Methods

### Tissue samples

A total of 283 patients with invasive breast cancer who underwent surgical treatment in the Department of Thyroid and Breast Surgery of Shanghai Tenth People's Hospital of Tongji University (Shanghai, China) from June 2017 to June 2022 were selected as the study objects. Inclusion criteria: female patients diagnosed with concurrent surgical treatment for the first time; the pathological examination was consistent with the diagnostic criteria of invasive breast cancer; the PIK3CA test was performed. Exclusion criteria: patients with other tumors or a history of tumors; patients with heart failure or dialysis; patients with abnormal mental state; pregnant or lactating patients; patients with incomplete clinical and follow-up data or missing follow-up.

All patients provided informed consent and the study was approved by the Ethics Committee for Clinical Research of Shanghai Tenth People’s Hospital. All patients had breast cancer specimens taken from surgical specimens. This study was reviewed and approved by the ethics committee of Shanghai Tenth People’s Hospital (approved number: SHSY-IEC-4.1/21-298/01) and conducted in accordance with the Declaration of Helsinki. This research is an observational study and follows the Strengthening the Reporting of Observational Studies in Epidemiology (STROBE).

### Data collection

Clinical data were obtained by collecting patient electronic medical record information, including the patient's age, BMI, basic disease history, menstrual history, lesion location, tumor size, metastasis of lymph nodes, tumor pathological grade and clinical stage, preoperative neutrophil and lymphocyte count, etc. ER, Progesterone Receptor (PR), HER2, and cell proliferation nuclear antigen-67 (Ki-67) were detected by immunohistochemistry of biopsy specimens. The specimens were Fluorescence In Situ Hybridization (FISH) using a PathVysion HER2 DNA probe kit.

### DNA extraction and PCR

Therascreen PIK3CA mutation assay the Therascreen® PIK3CA RGQ PCR Kit is a real-time qualitative PCR test for the detection of 11 mutations in PIK3CA (exon 9: E542K, E545D and E545K; exon 20: H1047L and H1047R) using genomic DNA extracted from formalin-fixed, paraffin-embedded breast tumor tissue.

### Follow-up

The patients were followed up by outpatient follow-up or telephone follow-up, and the information on local recurrence and distant metastasis was recorded. This study followed up the patients from postoperative to death or the end of follow-up, and the follow-up time ended on June 31, 2022. The follow-up time of 283 patients was 3- to 60-months. Disease Free Survival (DFS) was defined as the time from the first day after surgery to disease progression or the last follow-up, and DFS for PIK3CA mutant and wild-type patients were calculated and compared.

### Statistical analysis

SPSS 24.0 statistical software was used to analyze all the data. The mutation rate of PIK3CA was expressed as a percentage, and χ^2^ test was used for comparison between groups. Kaplan-Meier was used to draw the survival curve to analyze the influence of PIK3CA status on the prognosis of patients, and the Log-rank test was used to compare the recurrence rate between groups. The predictive factors of recurrence were screened by multivariate Logistic regression analysis, and the nomogram prediction model was established by the rms program package. The Consistency index (C-index) is calculated using the rms package. The calibration curve and Receiver Operator Characteristic Curve (ROC curve) were plotted, and the predictive efficiency of the area under the ROC Curve (AUC) was calculated; p < 0.05 indicates that the results have a statistical difference.

## Results

### Type and frequency of PIK3CA mutation

**Figure 1:**
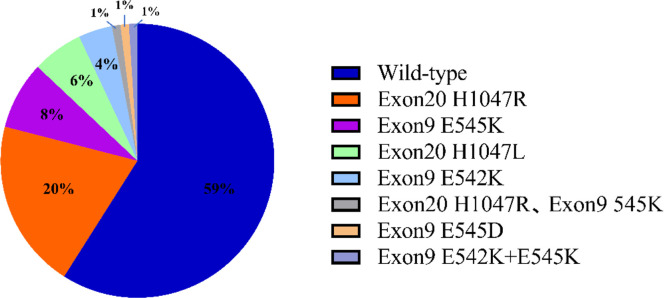
PIK3CA mutation profiles in a cohort of 283 breast cancer patients: Pie charts showing the types and frequencies of mutation detected.

A total of 116 PIK3CA mutations were detected in 283 invasive breast cancer tissue samples, representing a mutation rate of 41%. The PIK3CA test included the E542K, E545K, E545D, H1047L, and H1047R loci in exons 9 and 20. Among them, there were 72 cases with exon 20 mutation, accounting for 62.1% of the total mutation. There were 40 patients with exon 9 mutation, accounting for 34.5% of the total mutations. In addition, 2 patients had simultaneous mutations in exon 9 and exon 20, and 2 patients had simultaneous mutations in exon 9, E542K and E545K (Fig. 1).

### Relationship between PIK3CA mutation and clinicopathological features

Compared with wild-type PIK3CA tumors, PIK3CA-mutated tumors were significantly more prevalent in the upper outer quadrant of the breast and were more commonly associated with Invasive ductal carcinoma. Furthermore, these mutant tumors were typically identified at a later clinical stage, had a greater propensity for perineural invasion, and featured an elevated Neutrophil-to-Lymphocyte Ratio (NLR). All these correlations were statistically significant (p < 0.05, Table 1).

**Table 1:** Relationship between PIK3CA mutation and clinicopathologic features in breast cancer tissues.

Clinicopathological features	PIK3CA wild-type (n = 167)	PIK3CA mutant-type (n = 116)	p
Age [years, n (%)]			
< 50	50 (29.9%)	24 (26.1%)	0.082
≥ 50	117 (70.1%)	92 (73.9%)	
BMI [kg/m^2^, n (%)]			
< 24	144 (86.2%)	100 (86.2%)	0.996
≥ 24	23 (13.8%)	16 (13.8%)	
Menopausal status [n (%)]			
Post-menopause	117 (70.1%)	86 (74.1%)	0.454
Pre-menopause	50 (29.9%)	30 (25.9%)	
Hypertension [n (%)]			
Yes	38 (22.8%)	33 (28.4%)	0.277
No	129 (77.2%)	83 (71.6%)	
Diabetes mellitus [n (%)]			
Yes	19 (11.3%)	15 (12.9%)	0.693
No	148 (88.7%)	101 (87.1%)	
Tumor location [n (%)]			
Left breast	90 (53.9%)	57 (49.1%)	0.495
Right breast	79 (46.1%)	59 (50.9%)	
Tumor quadrants [n (%)]			
Outer upper	86 (51.5%)	73 (62.9%)	0.035
Outer lower	34 (20.3%)	10 (8.6%)	
Inner upper	36 (21.6%)	23 (19.9%)	
Inner lower	11 (6.6%)	10 (8.6%)	
Number of tumors [n (%)]			
Single	150 (89.8%)	109 (94.0%)	0.218
More	17 (10.2%)	7 (6.0%)	
Histological subtypes [n (%)]			
Invasive ductal carcinoma	141 (84.4%)	109 (94.0%)	0.013
Invasive lobular carcinoma	7 (4.2%)	0 (0.0%)	
Others	20 (11.4%)	7 (6.0%)	
Grade [n (%)]			
1	3 (1.8%)	5 (4.3%)	0.122
2	90 (53.9%)	50 (43.1%)	
3	74 (44.3%)	61 (52.6%)	
T stage [n (%)]			
T1~T2	166 (99.4%)	114 (98.3%)	0.600
T3~T4	1 (0.6%)	2 (1.7%)	
N stage [n (%)]			
N0	111 (66.5%)	80 (69.0%)	0.785
N1	29 (17.4%)	22 (19.0%)	
N2	18 (10.8%)	10 (8.6%)	
N3	9 (5.3%)	4 (3.4%)	
Clinical stage [n (%)]			
I	103 (61.7%)	53 (45.7%)	0.005
II	39 (23.4%)	48 (41.4%)	
III	25 (14.9%)	15 (12.9%)	
Vascular cancer thrombus [n (%)]			
Yes	46 (27.5%)	34 (29.3%)	0.746
No	121 (72.5%)	82 (70.7%)	
Perineural invasion [n (%)]			
Yes	11 (6.6%)	29 (25%)	0.000
No	156 (93.4%)	87 (75%)	
Inflammatory marks [n (%)]			
NLR	2.36 (0.94‒8.10)	2.63 (0.13‒16.25)	0.013
ER [n (%)]			
Positive	116 (69.5%)	88 (75.9%)	0.238
Negative	51 (30.5%)	28 (24.1%)	
PR [n (%)]			
Positive	94 (56.3%)	74 (63.8%)	0.206
Negative	73 (43.7%)	42 (36.2%)	
HER2 [n (%)]			
Positive	53 (31.7%)	25 (21.6%)	0.059
Negative	114 (68.3%)	91 (78.4%)	
Ki-67 [n (%)]			
≤ 14	48 (28.7%)	39 (33.6%)	0.382
> 14	119 (71.3%)	77 (66.4%)	

### PIK3CA mutations in different subtypes of breast cancer

**Figure 2:**
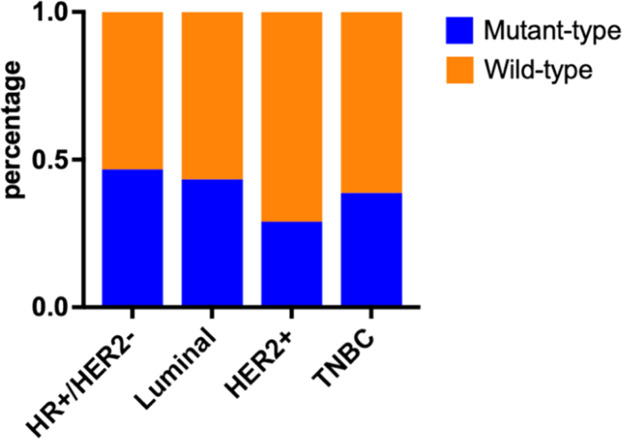
PIK3CA mutations in different molecular subtypes of breast cancer.

HR+/HER2- breast cancer patients had the highest probability of PIK3CA mutation (46.7%), and HER2+ breast cancer patients had the lowest probability of PIK3CA mutation (29.0%), but the difference was not statistically significant compared with other subtypes of breast cancer patients (p > 0.05, Fig. 2).

### Univariate analysis of poor prognosis of breast cancer

Compared with the non-recurrence group, patients in the recurrence group had higher BMI, more masses, more non-luminal types, a tendency to grade 3 pathology, advanced clinical stage, a higher risk of lymph node metastasis, positive vascular cancer thrombus, positive tumor perineural invasion, higher NLR, more positive HER2, and more common Ki-67 > 14%. All of these features were statistically significant (p < 0.05, Table 2).

**Table 2:** Univariate analysis of postoperative recurrence in breast cancer patients.

Clinicopathological features	Recurrence (n = 47)	Non-Recurrence (n = 236)	p
Age [years, n (%)]			
< 50	14 (29.8%)	60 (25.4%)	0.534
≥ 50	33 (70.2%)	176 (74.6%)	
BMI [kg/m^2^, n (%)]			
< 24	26 (55.3%)	218 (92.4%)	0.000
≥ 24	21 (44.7%)	18 (7.6%)	
Menopausal status [n (%)]			
Post-menopause	36 (76.6%)	167 (70.8%)	0.417
Pre-menopause	11 (23.4%)	69 (29.2%)	
Hypertension [n (%)]			
Yes	13 (27.7%)	58 (24.6%)	0.656
No	34 (72.3%)	178 (75.4%)	
Diabetes mellitus [n (%)]			
Yes	3 (6.3%)	31 (13.1%)	0.194
No	44 (93.7%)	205 (86.9%)	
Tumor location [n (%)]			
Left breast	21 (42.9%)	126 (53.4%)	0.179
Right breast	28 (57.1%)	110 (46.6%)	
Tumor quadrants [n (%)]			
Outer upper	33 (70.2%)	126 (53.4%)	0.051
Outer lower	7 (14.9%)	37 (15.7%)	
Inner upper	7 (14.9%)	52 (22.0%)	
Inner lower	0 (0.0%)	21 (8.9%)	
Number of tumors [n (%)]			
Single	38 (80.9%)	221 (93.7%)	0.004
More	9 (19.1%)	15 (6.3%)	
Histological subtypes [n (%)]			
Invasive ductal carcinoma	43 (91.5%)	207 (87.7%)	0.080
Invasive lobular carcinoma	3 (6.4%)	4 (1.7%)	
Others	2 (2.1%)	25 (10.6%)	
Grade [n (%)]			
1	1 (2.1%)	7 (3.0%)	0.019
2	15 (31.9%)	125 (53.0%)	
3	31 (66.0%)	104 (44.0%)	
T stage [n (%)]			
T1ཞT2	45 (95.7%)	235 (99.6%)	0.073
T3ཞT4	2 (4.3%)	1 (0.4%)	
N stage [n (%)]			
N0	12 (25.5%)	179 (75.8%)	
N1	6 (12.8%)	45 (19.1%)	0.000
N2	17 (36.2%)	11 (4.7%)	
N3	12 (25.5%)	1 (0.4%)	
Clinical stage [n (%)]			
I	5 (11.4%)	118 (50.0%)	0.000
II	14 (29.8%)	96 (40.7%)	
III	28 (58.8%)	22 (9.3%)	
Vascular cancer thrombus [n (%)]			
Yes	20 (42.6%)	60 (25.4%)	0.015
No	27 (57.4%)	176 (74.6%)	
Perineural invasion [n (%)]			
Yes	20 (42.6%)	20 (8.5%)	0.000
No	27 (57.4%)	216 (91.5%)	
Inflammatory marks [n (%)]			
NLR	3.31 (1.26‒13.98)	1.40 (0.13‒16.25)	0.013
PIK3CA [n (%)]			
Wild-type	31 (66.0%)	136 (57.6%)	0.289
Mutated	16 (34.0%)	100 (42.4%)	
Molecular typing [n (%)]			
Luminal	30 (63.8%)	173 (73.3%)	0.000
HER2+	13 (27.7%)	18 (7.6%)	
TNBC	4 (8.5%)	45 (19.1%)	
ER [n (%)]			
Positive	30 (63.8%)	174 (73.7%)	0.167
Negative	17 (36.2%)	62 (26.3%)	
PR [n (%)]			
Positive	26 (55.3%)	142 (60.2%)	0.536
Negative	21 (44.7%)	94 (39.8%)	
HER2 [n (%)]			
Positive	19 (40.4%)	59 (25.0%)	0.031
Negative	28 (59.6%)	177 (75.0%)	
Ki-67 [n (%)]			
≤ 14	7 (14.9%)	80 (33.9%)	0.010
> 14	40 (85.1%)	156 (66.1%)	

### Multivariate analysis of poor prognosis of breast cancer

**Figure 3:**
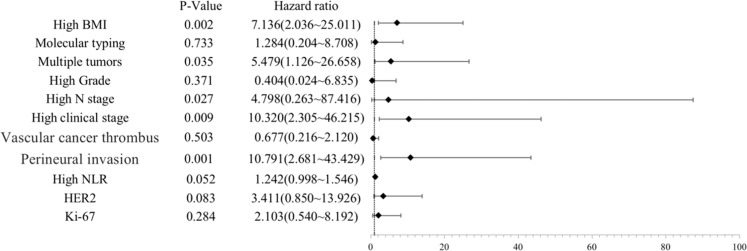
Multivariate logistic regression analysis of postoperative recurrence of breast cancer patients forest plot.

The indicators with statistically significant differences between groups in the univariate analysis were taken as independent variables, and the patients' recurrence (no recurrence = 0, recurrence = 1) was taken as dependent variables for multivariate Logistic regression analysis. The results showed that high BMI, multiple masses, lymph node metastasis, advanced clinical stage and tumor perineural invasion were independent risk factors for postoperative recurrence of breast cancer patients (p < 0.05, Fig. 3).

### Prediction model building

**Figure 4:**
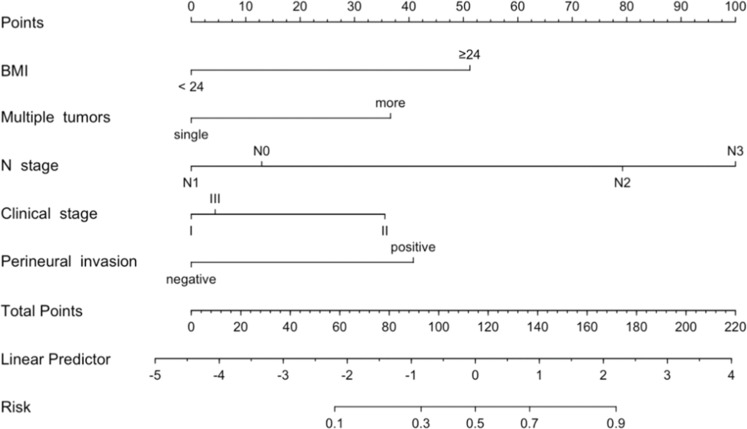
An established nomogram for predicting postoperative recurrence of breast cancer patients.

Independent predictors in multivariate Logistic regression analysis were used as predictors to construct a nomogram model to predict the risk of postoperative recurrence of breast cancer patients. Each factor and score scale in the column diagram corresponds to the size of the factor's ability to predict postoperative recurrence of breast cancer patients (Fig. 4).

### Model evaluation

**Figure 5:**
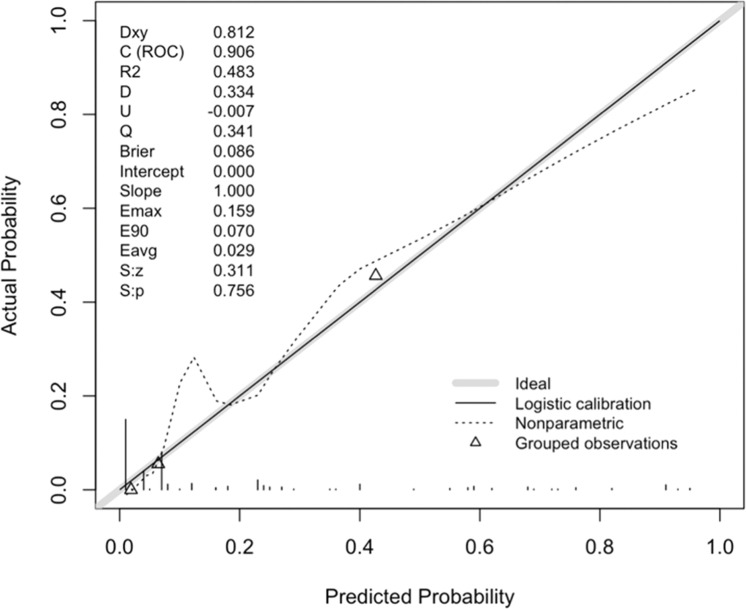
Calibration curve validation diagram for the nomogram model.

**Figure 6:**
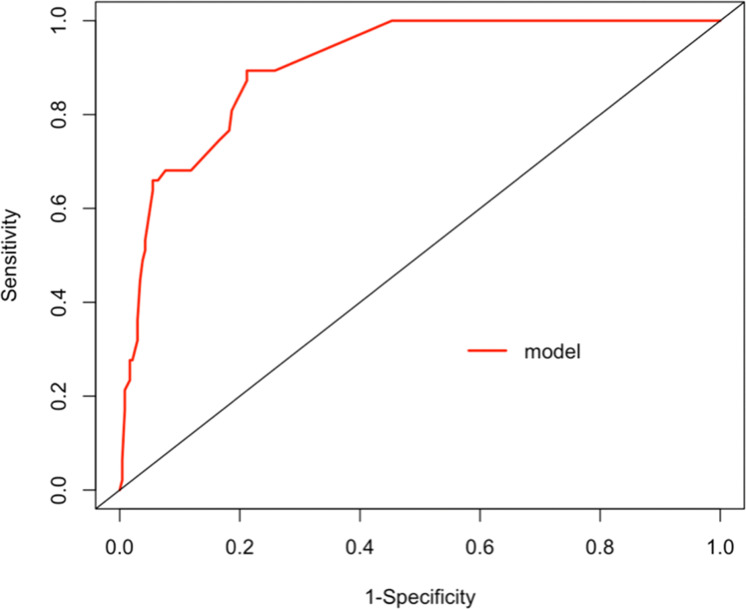
ROC curve validation diagram for the nomogram model.

A repeated sampling method was used to verify the nomogram model internally. The results showed that the C-index of the nomogram model was 0.906 (95% CI: 0.867∼0.944), and the calibration curve fitted well with the ideal curve (Fig. 5). The ROC curve showed that the AUC was 0.906 (95% CI: 0.867∼0.945), and the model had a high degree of differentiation (Fig. 6).

### Survival of patients with different PIK3CA status

**Figure 7:**
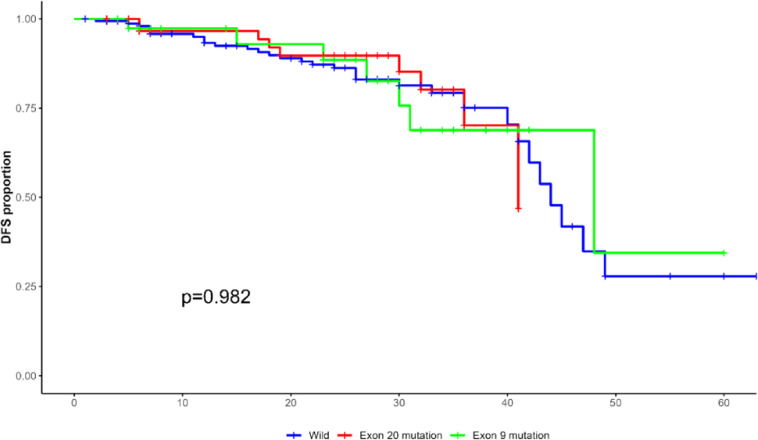
Survival curve analysis of PIK3CA in different states.

Recurrence of the disease was observed in 47 patients (16.6%), and the postoperative recurrence rate was 13.8% (16/116) and 18.6% (31/167) in patients with PIK3CA mutation and wild-type breast cancer, respectively. The DFS of breast cancer patients with PIK3CA exon 20 mutation, exon 9 mutation, and wild-type were 22.5-months, 32-months and 24-months, respectively, but the difference was not statistically significant (χ^2^ = 0.347, p = 0.982, Fig. 7).

Due to the heterogeneity of the sample, the authors conducted a stratified analysis for patients across different clinical stages. The findings of the present study are depicted in Supplementary Figures 1A, 1B, and 1C, which correspond to clinical stages I, II, and III, respectively. This analysis indicates that within each clinical stage, the impact of PIK3CA mutation status on DFS is not statistically significant (p > 0.05).

## Discussion

**Figure 8:**
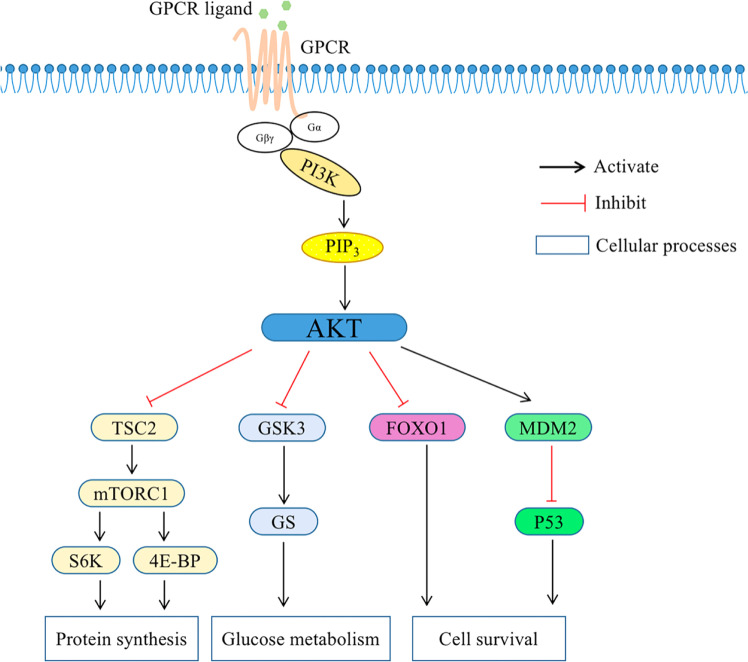
Schematic representation of the PI3K/AKT/mTOR signaling and its main components. AKT phosphorylates a large number of downstream effector proteins, including mTORC1, FOXO1, GSK3 and MDM2. These pathways regulate diverse cellular processes, including protein synthesis, cell survival, proliferation, glucose metabolism, apoptosis, DNA repair, and genome stability. GPCR, G Protein-Coupled Receptor; PIP3, Phosphatidylinositol-3,4,5-trisphosphate; mTORC1, Mammalian Target of Rapamycin Complex-1; TSC2, Tuberous Sclerosis Complex-2; GSK3, Glycogen Synthase Kinase-3; GS, Glycogen Synthase; FOXO1, Forkhead box O1; S6K, S6 Kinase; 4E-BP, 4E-Binding Protein; MDM2, Mouse Double Minute 2 homolog.

Breast cancer remains the most common malignancy in women,^10^ with complex pathogenesis involving genetic, environmental, and immunological factors.^11^ Genetic mutations, such as those in PIK3CA, play a critical role in breast cancer progression, pathological classification, and prognosis.^12^, ^13^, ^14^ PIK3CA, located on chromosome 3, encodes the p110α catalytic subunit of PI3K and is frequently mutated in breast cancer, particularly in HR+/HER2- subtypes.^15^ PIK3CA mutation induces the continuous Activation of Protein Kinase B (PKB/AKT) through the PI3K/AKT pathway, leading to the growth and transformation of fibroblasts and mammary epithelial cells and inhibiting apoptosis, which is closely related to the occurrence and development of breast cancer (Fig. 8).

In this study, the authors identified a 41% PIK3CA mutation rate among Chinese breast cancer patients, with exon 20 mutations predominating (62.1%). HR+/HER2- patients exhibited the highest mutation frequency (46.7%), consistent with prior reports. However, no PIK3CA mutations were detected in invasive lobular carcinoma cases, and HER2+ breast cancer showed the lowest mutation rate, potentially due to sample size and ethnic differences. These findings highlight the importance of PIK3CA mutations in breast cancer subtyping but suggest further validation is needed to clarify their prognostic significance.

In addition, the effects of PIK3CA mutation on the prognosis of different subtypes and different periods of breast cancer are also different. Lee MH et al. isolated DNA from the normal and tumor tissues of 128 patients with invasive breast cancer, and analyzed the mutation and expression of PIK3CA, and found that PIK3CA mutation and expression were significantly correlated with Luminal A breast cancer.^16^ Zardavas D et al. found that in early HR+/HER2- breast cancer, PIK3CA mutation suggested a good prognosis for patients.^17^ Takeshita T et al. confirmed that early TNBC patients with PIK3CA mutations had a better prognosis than those with PIK3CA wild-type.^18^ Mosele F et al. showed that PIK3CA mutation suggested a poor prognosis for patients with metastatic HR+/HER2- breast cancer and a good prognosis for patients with metastatic TNBC.^19^ Similarly, Wang et al. found PIK3CA mutation may be associated with a worse prognosis.^20^ However, other studies have shown that the mutation of PIK3CA has no significant relationship with the prognosis of breast cancer patients.^10^^,^^21^ In addition, mutations in different gene loci of PIK3CA may also serve as prognostic indicators for patients. The researchers found that patients with mutations in exon 9 of PIK3CA have worse prognosis than those with mutations in exon 20.^22^ In this study, the authors found that PIK3CA mutation is closely related to tumor quadrants, histological subtypes, advanced clinical stage, tumor perineural invasion and high NLR. Recurrence of the disease was observed in 47 patients (16.6%), and the postoperative recurrence rate was 13.8% (16/116) and 18.6% (31/167) in patients with PIK3CA mutation and wild-type breast cancer, respectively. The DFS of breast cancer patients with PIK3CA exon 20 mutation, exon 9 mutation and wild-type were 22.5-months, 32-months and 24-months, respectively. These results suggested that breast cancer patients with PIK3CA exon 9 mutation had the best prognosis, while patients with PIK3CA exon 20 mutation had lower DFS than those with wild type, but the difference was not statistically significant (χ^2^ = 0.347, p > 0.05).

The relationship between PIK3CA mutation and clinicopathological features of breast cancer patients is still controversial. Lv W et al. confirmed that PIK3CA mutation status was related to age, histopathologic type, pathological grade, ER positive, PR positive, molecular subtype and family history.^22^ Wu H et al. found that PIK3CA mutation was associated with ER positive, PR positive, low Ki-67 marker index and HER2 subtype.^23^ In contrast, Ben Rekaya M et al. found that age, histological grade, ER, PR, HER2, and molecular typing were not associated with PIK3CA mutation.^24^ In the present study, no correlation was found between ER, PR, HER2, Ki-67, tumor size and PIK3CA mutations. More studies with multiple centers and large sample sizes are needed to further clarify these differences.

The status of PIK3CA may influence the efficacy of targeted therapy and endocrine therapy in breast cancer patients. Some studies have found that the pathological complete response rate of HER2-positive breast cancer patients with wild-type PIK3CA is significantly higher than that of patients with mutant type.^25^^,^^26^ Since the efficacy of HER2-positive breast cancer patients with PIK3CA mutation receiving trastuzumab treatment is significantly reduced, and the overall survival is significantly shortened, therefore, HER2-positive breast cancer patients with PIK3CA mutation may develop resistance to chemotherapy and anti-HER2 targeted therapy, and more studies are needed to clarify the mechanism.^27^, ^28^, ^29^ For HR+/HER2- breast cancer, endocrine therapy is the most important treatment, but Huang D et al. found that PIK3CA mutation can lead to fulvestrant resistance.^30^ In order to improve the efficacy of endocrine drugs, PI3K inhibitors are used clinically. To date, alpelisib is the only specific PI3K inhibitor approved by the FDA for the treatment of breast cancer. Andre F et al. divided 341 patients with advanced breast cancer with PIK3CA mutation into two groups and found that DFS in the alpelisib combined with fulvestrant group was significantly higher than that in the placebo combined with fulvestrant group. In addition, significant benefits were also demonstrated in terms of the overall response rate and the clinical benefit rate.^31^

This study has the following limitations: The number of samples included in this study is insufficient and cannot reflect the overall level of breast cancer oncogene mutations due to regional factors. The authors will collect more breast cancer cases from more regions in the subsequent study for multi-center research. In addition, in future work, the authors will replace more accurate combined sequencing methods for the detection of unknown mutations, in order to reduce errors and provide more accurate data support for clinical diagnosis and treatment and prognosis assessment of breast cancer.

## Conclusions

This study analyzed 283 Chinese women with invasive breast cancer and found a PIK3CA mutation rate of 41%, with exon 20 mutations being the most common (62.1%). PIK3CA mutations were significantly associated with tumor quadrant distribution, histological subtype, advanced clinical stage, perineural invasion, and high NLR, but no significant impact on DFS was observed. Multivariate analysis identified high BMI, multiple tumors, lymph node metastasis, advanced stage, and perineural invasion as independent risk factors for postoperative recurrence. While PIK3CA mutations are implicated in breast cancer progression, their prognostic utility requires validation through larger multicenter studies. Future research should focus on subtype-specific roles and therapeutic implications, particularly in HR+/HER2- subgroups.

## Authors’ contributions

Juhang Chu: Methodology; data curation; software; writing-original draft preparation.

Luyao Huang: Data curation; validation.

Yaru Wang: Formal analysis; visualization.

Mingping Qian: Conceptualization; project administration.

## Declaration of competing interest

The authors declare no conflicts of interest.

## Appendix Supplementary materials

Image, application 1
