# Supplementary figures and images for: Clinicopathological and prognostic analysis of PIK3CA mutated invasive breast cancer in Chinese women
Source: Clinics (Sao Paulo). 2025 Jun 4;80:100702. doi: 10.1016/j.clinsp.2025.100702 (PMC12171758; doi:10.1016/j.clinsp.2025.100702)

**CLINICS-D-24-00367_ Supplementary Material**

**Supplementary Figure 1** Findings of the present study.


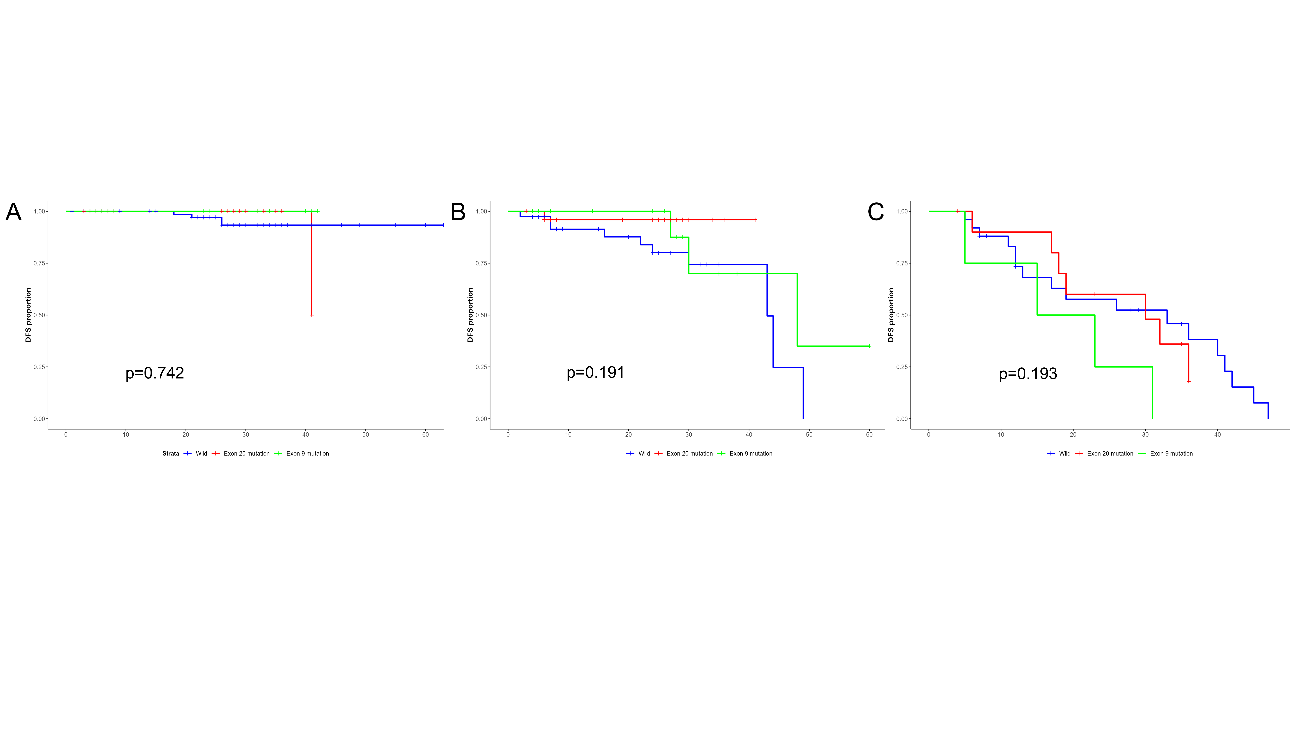

Supplement: Supplementary file 1 [file mmc1.docx]
